# Measurement of Reverse Cholesterol Transport Pathways in Humans: In Vivo Rates of Free Cholesterol Efflux, Esterification, and Excretion

**DOI:** 10.1161/JAHA.112.001826

**Published:** 2012-08-24

**Authors:** Scott Turner, Jason Voogt, Michael Davidson, Alex Glass, Salena Killion, Julie Decaris, Hussein Mohammed, Kaori Minehira, Drina Boban, Elizabeth Murphy, Jayraz Luchoomun, Mohamad Awada, Richard Neese, Marc Hellerstein

**Affiliations:** 1 KineMed, Inc, Emeryville, CA (S.T., J.V., A.G., S.K., J.D., H.M., E.M., J.L., M.A.); 2 Radiant Research, Chicago, IL (M.D.); 3 Department of Nutritional Sciences and Toxicology, University of California at Berkeley, Berkeley, CA (K.M., R.N., M.H.); 4 Department of Medicine, Division of Endocrinology and Metabolism, University of California at San Francisco, San Francisco, CA (D.B., M.H.)

**Keywords:** cholesterol efflux, esterification, reverse cholesterol transport, isotope labeling, stable, sterol excretion

## Abstract

**Background:**

Reverse cholesterol transport from peripheral tissues is considered the principal atheroprotective mechanism of high-density lipoprotein, but quantifying reverse cholesterol transport in humans in vivo remains a challenge. We describe here a method for measuring flux of cholesterol though 3 primary components of the reverse cholesterol transport pathway in vivo in humans: tissue free cholesterol (FC) efflux, esterification of FC in plasma, and fecal sterol excretion of plasma-derived FC.

**Methods and Results:**

A constant infusion of [2,3-^13^C_2_]-cholesterol was administered to healthy volunteers. Three-compartment SAAM II (Simulation, Analysis, and Modeling software; SAAM Institute, University of Washington, WA) fits were applied to plasma FC, red blood cell FC, and plasma cholesterol ester ^13^C–enrichment profiles. Fecal sterol excretion of plasma-derived FC was quantified from fractional recovery of intravenous [2,3-^13^C_2_]-cholesterol in feces over 7 days. We examined the key assumptions of the method and evaluated the optimal clinical protocol and approach to data analysis and modeling. A total of 17 subjects from 2 study sites (n=12 from first site, age 21 to 75 years, 2 women; n=5 from second site, age 18 to 70 years, 2 women) were studied. Tissue FC efflux was 3.79±0.88 mg/kg per hour (mean ± standard deviation), or ≍8 g/d. Red blood cell–derived flux into plasma FC was 3.38±1.10 mg/kg per hour. Esterification of plasma FC was ≍28% of tissue FC efflux (1.10±0.38 mg/kg per hour). Recoveries were 7% and 12% of administered [2,3-^13^C_2_]-cholesterol in fecal bile acids and neutral sterols, respectively.

**Conclusions:**

Three components of systemic reverse cholesterol transport can be quantified, allowing dissection of this important function of high-density lipoprotein in vivo. Effects of lipoproteins, genetic mutations, lifestyle changes, and drugs on these components can be assessed in humans. **(*J Am Heart Assoc*. 2012;1:e001826 doi: 10.1161/JAHA.112.001826.)**

## Introduction

The regulation of cellular cholesterol homeostasis is crucial for membrane function and cell survival and is maintained by multiple mechanisms, including control of uptake, synthesis, storage, and efflux. Compared to the pathways of cellular uptake and de novo synthesis of cholesterol, however, less information exists about the control of flux though pathways that remove cholesterol from cells and from the whole organism,^1–2^ particularly in humans.^3–5^ These pathways collectively have been termed *reverse cholesterol transport* (RCT).

RCT is postulated to play a fundamental role in cholesterol homeostasis and distribution among tissues^5^ and thereby in the development and reversal of atherosclerosis.^6–7^ The atheroprotective effects of high-density lipoprotein cholesterol (HDL-C) in both human and animal studies often have been attributed to its central role in the RCT pathway,^8^ although other antiatherogenic effects of HDL also have been proposed.^9–10^ The functional significance of whole-body or macrophage-specific RCT pathways in atherogenesis is not definitively proven.^11^ It is clear, however, that systemic factors, such as plasma acceptor capacity for efflux of free cholesterol (FC) from tissues, capacity for plasma esterification of FC and transfer of cholesterol ester (CE) from HDL to low-density lipoprotein particles, as well as hepatobiliary/intestinal excretion of sterols in the feces, can influence both local and whole-organismal RCT fluxes. The RCT pathway remains a target for pathophysiological inquiry and therapeutic intervention.^3,12–13^

Systemic RCT in the whole body can be conceived as comprising 3 primary components: FC efflux from tissues into the extracellular space (tissue cholesterol efflux [TCE]), cholesterol esterification and transport in the plasma compartment, and excretion from the body into the feces as neutral sterols (NS) and bile acids (BA) (fecal sterol excretion [FSE]).^7,14–15^ The first step in RCT is the movement of FC from cell membranes to HDL in the extracellular space.^5,16^ This is considered to be a bidirectional process whereby HDL particles both deliver and accept FC.^5^ This function of HDL has been well studied in cell culture.^5,15^ In vivo, the movement of FC between cell membranes and HDL can generate directional (net) flux by several mechanisms, including ATP-utilizing, energy-dependent transport^16–17^; lecithin:cholesterol acyltransferase (LCAT)–catalyzed esterification of FC^14^; or physical removal by other tissues of cholesterol delivered through the circulation, with regeneration of cholesterol-poor acceptor particles.^18^ All of these mechanisms can drive net removal of cholesterol from developing atherosclerotic lesions or other cholesterol-rich tissues after surface transfer of FC from vessel wall cell membranes to HDL particles. The second component of RCT, esterification of FC to CE on plasma lipoprotein particles, is catalyzed by the enzyme LCAT and is an energy-dependent, irreversible step.^19^ LCAT activity was a central element in the original concept of centripetal flux of cholesterol away from tissues and to the liver, or RCT.^14^ In animals that express CE transfer protein, CE can exit the circulation into tissues, including the liver, either on HDL-C or low-density lipoprotein cholesterol particles.^20–21^ LCAT is under complex physiological control; substrate activation of LCAT is observed ex vivo when FC effluxes into lipoprotein particles, for example. The third component of RCT is excretion of plasma cholesterol from the body into fecal sterols, which involves complex mechanisms in the liver and intestine^22^ and results in loss of cholesterol from the whole organism.

In the present article, we describe a method that uses a single tracer administration for measuring these 3 primary components of the systemic RCT pathway: TCE, plasma FC esterification, and FSE. Our primary objectives were to test the key assumptions of the method in humans and to evaluate the optimal clinical protocol and approach to data analysis and modeling. Parts of this work have been presented previously in abstract form.^23–24^

## Methods

After the description of 3 compartment models of whole-body cholesterol metabolism,^25-27^ several groups have measured plasma cholesterol dynamics in humans though detailed analysis of multicompartmental decay curves of radiolabeled cholesterol.^28–31^ Their work, most notably that of Schwartz and colleagues,^29,31^ established several points. First, rapid equilibration of FC occurs within the plasma lipoprotein compartment as well as in hepatobiliary FC pools within hours. Second, entrance of the vast majority of cholesterol from tissues into blood is in the form of FC. Third, almost all (>95%) of FC enters the plasma compartment on HDL particles. These findings suggested to us that application of a labeled FC constant infusion approach^32^ can capture the FC flux rate between unlabeled pools in tissues and the rapidly labeled FC pool, which includes plasma lipoproteins, and can therefore be sampled easily. Seen in this manner, the movement of unlabeled FC from slow-turnover pools in extrahepatic tissues dilutes the infused labeled pool in plasma and liver, while labeled plasma FC leaves the circulatory pool for these tissues. These processes represent FC efflux from extrahepatic tissues into the plasma compartment (TCE) and FC influx into tissues, respectively. Plasma FC also can be esterified by LCAT to CE, which then could exit the circulation. At metabolic steady state for plasma FC, TCE must equal (ie, be balanced by) FC influx into tissues plus FC esterification. Quantification of labeled fecal sterols (FSE) completes the characterization of RCT pathway fluxes.

To measure these processes, we modeled plasma FC and CE enrichments during constant infusions of [2,3-^13^C_2_]-cholesterol and the recovery of labeled fecal NS and BA after the infusions in human subjects.^33^

### Plasma Cholesterol Flux Model

**Figure 1.**
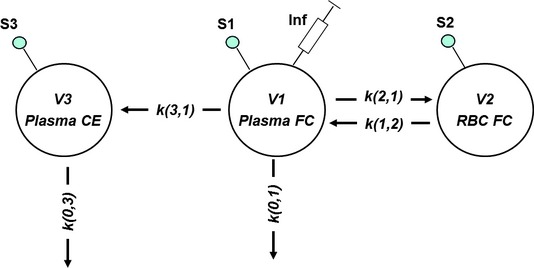
Multicompartment model of fluxes of FC. SAAM II model of cholesterol fluxes in and out of the plasma FC pool (V1), RBC FC (V2), plasma CE (V3), and rapid-exchange (mixing) pool in equilibrium with V1 (V4). k indicates rate constants 1/hour; s, sampling sites – data input into model; Infusion, site of constant infusion of FC. Parameters: R, infusion rate (mg/kg per hour); V1, plasma FC pool size (mg/kg body weight); V2, RBC FC pool size (mg/kg body weight); V3, plasma CE pool size (mg/kg body weight); k(0,1), rate constant for transfer of tracer out of plasma FC pool (hour^−1^); k(0,3), rate constant for transfer of tracer out of plasma CE pool (hour^−1^); k(3,1), rate constant for transfer of tracer from plasma FC to plasma CE pool (hour^−1^); k(1,2), rate constant for transfer of tracer from RBC FC pool to plasma FC pool (hour^−1^); k(2,1), rate constant for transfer of tracer from plasma FC pool to RBC pool (hour^−1^); and s_1_, s_2_, and s_3_ sampling sites, corresponding with V_1_, V_2_, and V_3_. R, V_2_, and V_3_ entered as fixed parameters into the model; others are calculated by SAAM II. Steady-state equations: Flux 1=k(0,1)×V1=flux of plasma FC out of V1 (mg/kg per hour); Flux 2=k(2,1)×V1=k(1,2)×V2=exchange flux between plasma FC and RBC FC (mg/kg per hour); Flux 3=k(0,3)×V3=k(3,1)×V1=flux of plasma FC to plasma CE pool (mg/kg per hour); Flux 1+Flux 3=TCE (mg/kg per hour).

A multicompartment model was used to determine the flux of FC into and out of plasma. Labeled FC ([2,3-^13^C_2_]-cholesterol, [^13^C cholesterol]) was infused into volunteers over a period of 24 or 32 hours, and the isotopic enrichment and the pool sizes of plasma and erythrocyte (red blood cell [RBC]) FC pools and CE pools were measured. Data were analyzed with SAAM II software (Simulation, Analysis, and Modeling; SAAM Institute, University of Washington, WA).^34^ The kinetic model is schematically illustrated in Figure 1. This model was designed to incorporate data from the major pools of cholesterol. V1 represents the rapidly exchanging cholesterol pools, which include the plasma FC pool and likely include but are not limited to the liver FC pool (Figure 1, V1).^29,31^ V2 corresponds to the erythrocyte FC pool and is measured. V3 represents plasma cholesterol-ester (CE) and also is measured. The size of pool V1 is determined by the SAAM fit. Furthermore, the size of V1+V2 should be similar to the rapidly miscible pool of FC identified by previous bolus-decay studies.^25,27,26^

The mass of FC present in RBCs (V2) was measured from the FC content of RBCs measured by chemical assay (milligrams FC/grams RBC) and hematocrit times blood volume (estimated as 7% of body weight). Isotopic enrichment profiles of RBC FC and plasma FC were used by the SAAM II fits to determine exchange flux between RBC FC and plasma FC.

Plasma esterification rate (LCAT flux) was measured from CE/FC enrichment time profiles. Esterification rate was calculated from the precursor–product relationship between plasma FC and CE isotopic enrichments^32^ and the plasma CE pool size by using SAAM II fits. CE pool size (V3) was measured by chemical assay (see below) as the difference between total and FC times plasma volume (see equation below). It should be noted that the LCAT flux can be determined from these data independently of the full model by using simple precursor–product calculations, which yield identical results.

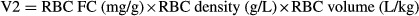
(1)

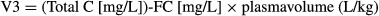
(2)

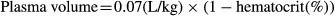
(3)

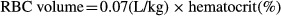
(4)

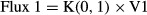
(5)

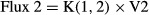
(6)

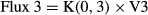
(7)

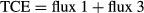
(8)

Fluxes between the plasma FC pool (V1) and V2 (RBC FC) represent reversible, or non-net flux, for labeled plasma FC, whereas flux 1, loss of FC from V1, and flux 3 (cholesterol esterification) are considered to be unidirectional during the timeframe of the study. In this model, TCE is a flux parameter of primary interest, representing the mobilization of FC from extrahepatic tissues into the rapid-turnover FC pool (V1) in plasma, and does not return during the course of the study. When cholesterol pool sizes are at steady state, TCE equals the flow into and out of the plasma FC pool and into tissues and plasma CE. Here, TCE is calculated as the sum of flux 1 and flux 3 (Figure 1).

The model assumes that (1) subjects are at metabolic steady state (ie, constant weight and total cholesterol concentrations), (2) each pool is at metabolic steady state over the course of the infusion study (ie, flux in = flux out), (3) esterification of plasma FC is irreversible, and (4) there is no direct removal of RBC FC to other parts of the system besides plasma FC. Inputs into the model are the masses of V2 and V3, the infusion rate of ^13^C cholesterol, the ^13^C cholesterol enrichments of FC sampled in V1 and V2, and ^13^C CE enrichments in V3. All other parameters are calculated by the model.

### Excretion Efficiency of Plasma Cholesterol Into Fecal Sterols

Total fecal excretion of sterols was measured from stool samples through the administration of ^2^H_4_-sitostanol (Sigma Aldrich) capsules, 3 mg 3 times a day for 10 days. Sterol excretion (excretion) of total NS and BA was calculated as^34^:

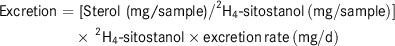


Total isotope recovery in stool over the 7-day period after [2,3-^13^C_2_]-cholesterol administration was determined from the NS and BA enrichments multiplied by total sterol excretion rates. NS and BA enrichments were determined by isotope ratio mass spectrometry. Preinfusion stool samples were used to determine baseline enrichments in each fraction, which were subtracted from postinfusion samples to determine ^13^C content arising from administered label. The ^2^H_4_-sitostanol excretion rate was assumed to equal the daily intake (9 mg/d). Total sterol excretion was calculated from the area under the curve of NS and BA multiplied by the average daily excretion of the subject.

Excretion efficiency of plasma cholesterol into fecal sterols was calculated as the ratio of total isotope recovery (summed over 7 days) in fecal NS or BA to the total [2,3-^13^C_2_]-cholesterol dose administered intravenously during the infusion.

### Materials

[2,3-^13^C_2_]-cholesterol (^13^C_2_-C) (99%) was purchased from Cambridge Isotope Labs (Andover, MA) and Isotec Inc (Miamisburg, OH). [5,6,22,23-^2^H_4_] sitostanol was purchased from Isotec Inc (Miamisburg, OH). NS standards were purchased from Steraloids (Newport, RI). Other reagents were from Sigma (St. Louis, MO). Ten percent Intralipid (Fresenius-Kabi, Uppsala, Sweden) was purchased from Baxter Inc (Deerfield, IL).

### Study Protocol

RCT studies were performed at San Francisco General Hospital, University of California at San Francisco General Clinical Research Center (San Francisco, CA) and Radiant Research (Chicago, IL).

All studies received approval from the University of California at San Francisco, Radiant Research, and Western IRB (Birmingham, AL) institutional review boards, and subjects gave written informed consent before participating. The infusate was prepared by a modification of the method described by Ostlund et al.^33^ Briefly, 200 mg of ^13^C_2_-cholesterol was dissolved into 13 mL of warm US Pharmacopeia-grade ethanol. This solution then was mixed slowly into 120 mL of 10% Intralipid (Fresenius-Kabi, Uppsala, Sweden) solution to a final concentration of 1.5 mg/mL ^13^C_2_-cholesterol.

At the Radiant Research site, subjects were recruited who were nonsmoking men and women, 21 to 75 years of age inclusive. Subjects on lipid-lowering therapy within 2 months before the study or with a known history of coronary heart disease, stroke, prior revascularization procedure, or peripheral vascular disease were excluded, as were those with diabetes mellitus. Other exclusions included baseline elevations in the ratio of aspartate transaminase to alanine transaminase >2× the upper limit of normal, fasting glucose levels ≥7.0 mmol/L (≥126 mg/dL), abnormal thyroid-stimulating hormone, or laboratory evidence of renal impairment. Baseline plasma lipids are shown in Table 1.

**Table 1.** Cholesterol Pool Sizes and Fasting Plasma Lipids of Participants

			Lipids, mg/dL			Pool Sizes, mg/kg		
Participants	M/F	Weight, kg	HDL-C	LDL-C	TG	V1	V2	V3
435	F	67	53	156	222	78	14	42
442	F	85	58	148	179	72	24	36
454	M	96	38	194	292	78	25	48
458	M	84	36	158	131	64	25	38
459	M	88	49	134	97	66	25	32
58	M	122	30	99	578	63	23	53
63	M	149	36	141	113	62	22	50
64	M	83	30	143	203	150	25	57
61	M	95	32	112	213	70	37	57
65	M	82	38	131	252	92	34	71
66	M	84	33	82	246	93	29	65
69	M	108	51	113	98	56	30	53
70	M	65	56	na	36	61	57	50
71	M	82	131	95	81	42	57	64
72	M	85	47	146	98	69	54	75
73	F	79	69	150	95	62	54	79
75	F	54	69	97	60	100	32	60
Mean		90	50	128	173	75	35	57
SD		23	26	30	136	26	13	13

Individual cholesterol pool sizes are given in milligrams per kilogram. M indicates male; F, female; LDL-C, low-density lipoprotein cholesterol; TG, triglycerides; V1, the compartment that includes FC in plasma lipoproteins as well as presumed liver and other rapidly equilibrated pools; V2, erythrocyte FC; V3, plasma CE; and na, not applicable.

Subjects were admitted to the clinical research center for a 26-hour in-clinic stay for stable isotope infusions. An early supper was served with ad libitum food intake allowed. After 5:00 pm, food and caloric-containing beverages were withheld. Subjects had one intravenous catheter placed for cholesterol infusion and one for blood draws. A stable isotope infusion of [^13^C_2_]-cholesterol mixed in 10% Intralipid and 10% ethanol was given, piggy-backed into normal saline over 24 hours (6 pm to 6 pm). A total of 200 mg of labeled cholesterol was given. Upon discharge, subjects were given ^2^H_4_-sitostanol to take, 3 mg orally 3 times daily with meals, to quantify fecal NS and BA excretion from casual stool samples as described previously.^35–36^ A single baseline stool sample was taken 3 days before the infusion, after which the subjects were given sitostanol for 10 days (day 3 through day 7 after infusion). Daily stool samples were taken, when available, for the 3 days before and 1 week after infusion; only days 1 to 7 after infusion were used for analysis. Twelve of the 17 subjects underwent the infusion protocol at the Radiant site.

At the San Francisco site, men and women age 18 to 70 years were eligible to participate in this study. Exclusion criteria included any serious medical illness or potentially confounding condition, recent weight loss or constitutional symptoms, alcohol or substance abuse, and inability to provide informed consent. Baseline plasma lipids are shown in Table 1. Subjects were admitted at 3:00 pm for 34 hours. After a low-fat meal at 5 pm, food was withheld. Subjects had one intravenous catheter placed for infusion of ^13^C_2_-cholesterol and one for blood draws. An intravenous infusion of ^13^C_2_-cholesterol, piggy-backed into normal saline (100 cc/h), was administered over 32 hours at a rate of 3.5 mL/h. Subjects were allowed food intake after 24 hours. Subjects had 5 mL of blood drawn into EDTA at regular intervals throughout the infusion. Immediately after drawing, blood was spun, plasma was separated, and the residual RBCs were washed twice with saline. Before admission, no diet recommendations were made or records collected. FSE was not measured in these subjects. Five of the 17 subject studies underwent the infusion protocol at the San Francisco site.

### Analysis of Cholesterol Metabolites

Plasma FC was extracted with ethanol-acetone, acetylated with toluene/pyridine/acetyl chloride, and dissolved in toluene for analysis by mass spectrometry. FC from RBCs was analyzed after homogenization with silicate beads and extraction in chloroform-methanol. For measurement of CE enrichment, extracted plasma FC and CE were first separated on an amino-propyl solid phase extraction cartridge, the fatty acid moiety of the CE was cleaved by methanolic HCl, and the resultant FC was acetylated.

Stool samples first were homogenized with an equal volume of water, and then NS and BA were extracted separately under basic and acidic conditions, respectively, in the presence of the internal standards 5-σ-cholestane and 5-β-cholanic acids. The BA extract was split into 2 fractions. The first, used for compositional analysis by flame ionization detection, was directly subjected to a 2-step derivatization: butylation with butanolic HCl, followed by silylation by N,O-Bis(trimethylsilyl)trifluoroacetamide-pyridine. The second half was further purified for mass spectrometric isotopic analysis with an octadecyl solid phase extraction cartridge, selectively eluting primarily deoxycholic acid with a 20% aqueous-methanol solution before butylation and silylation. The NS fraction was silylated directly for both compositional and isotope analysis.

Isotopic enrichments of ^13^C-cholesterol were measured by using gas chromatography/C-isotope ratio mass spectrometry (Thermo Finnegan MAT 253 IR-MS, Bremen, Germany). Enrichments were determined as atom percent excess by comparison of the unknown samples to a standard curve generated with gravitametrically prepared working laboratory standards with known enrichments. Molar percent excess is calculated as 14.5× or 15× atom percent excess for the acetyl or silyl derivative of cholesterol, respectively,^37^ and by 17× atom percent excess for the butyl-silyl derivative of deoxycholic acid (the correction factors account for dilution of labeled H-atoms in the sterol by unlableled H-atoms in the derivatizing moiety). Compositional analysis and excretion measurement of BA and NS were performed by gas chromatography/flame ionization detection with comparison to the internal standards and sitostanol.^35^ Gas chromatography peak areas of cholesterol, coprostanol, epicoprostanol, coprostan-3-one, and cholestanol were used to calculate NS mass. Gas chromatography peak areas of isolithocholic, isodeoxycholic, lithocholic, deoxycholic, cholic, chenodeoxycholic, ursodeoxycholic, and 7-ketolithocholic were used to calculate acidic sterol mass.

Total fecal excretion of sterols was measured from stool samples through the administration of ^2^H_4_‐sitostanol and “5,6,22,23‐^2^H_4_‐sitostanol” (Sigma Aldrich) capsules, 3 mg 3 times a day for 10 days. Sterol excretion (excretion) of NS or BA was calculated from the equation shown previously.

Total isotope recovery in stool over the 7-day period after [2,3-^13^C_2_]-cholesterol administration was determined from the NS and BA enrichments multiplied by total sterol excretion rates. NS and BA enrichments were determined by isotope ratio mass spectrometry. Preinfusion stool samples were used to determine baseline enrichments in each fraction, which were subtracted from postinfusion samples to determine ^13^C content arising from administered label. Total sterol excretion was calculated from the area under the curve of NS and BA multiplied by the average daily excretion of the subject.

Excretion efficiency of plasma cholesterol into fecal sterols was calculated as the ratio of total isotope recovery (summed over 7 days) in fecal NS or BA to the total [2,3-^13^C_2_]-cholesterol dose administered.

### Particle Size Analysis

Lipoprotein particle size analysis was performed on a subset of samples from subjects studied at the University of California San Francisco site with nuclear magnetic resonance by Liposcience Inc (Raligh, NC). Liposcience uses a nuclear magnetic resonance lipoprotein analysis method to measure lipoprotein subclass particle concentrations and average very-low-density lipoprotein, low-density lipoprotein, and HDL particle diameters. The particle concentrations of the different-sized lipoprotein subclasses in blood plasma are given by the measured amplitudes of the characteristic lipid methyl group nuclear magnetic resonance signals emitted.^38^

### Statistical Analysis

All data presented are mean±standard deviation (SD). Nonparametric tests were performed in GraphPad Prisim 5 software (GraphPad Software, La Jolla, CA).

## Results

### Isotope Enrichment Profiles of Plasma FC During Constant Infusions of [^13^C_2_]-Cholesterol

**Figure 2.**
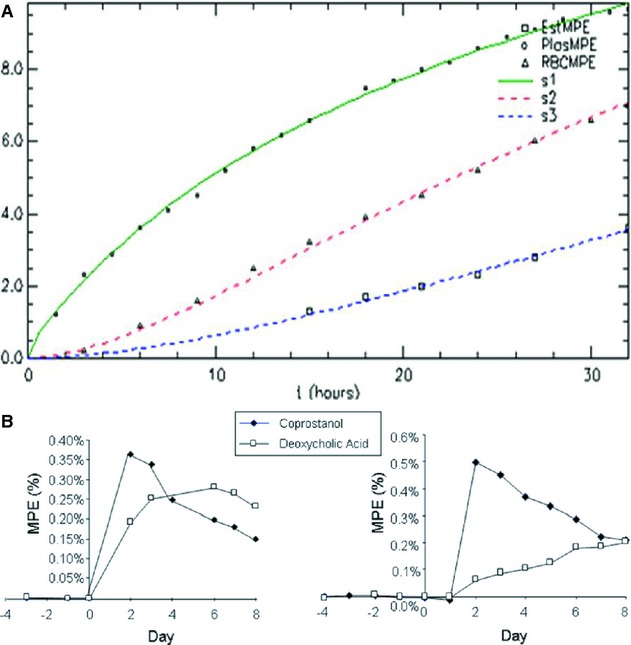
A, Example of a typical isotope enrichment profiles in plasma FC (circles), RBC FC (triangles) and CE (squares) in subject during a 32-hour isotope infusion. Solid lines represent the SAAM II–generated fits. B, Enrichment profiles in stool for a NS, coprostanol (closed), and a BA, deoxycholic acid (open). Infusion was on day zero, and enrichment was measured in daily stool collections, when available. Preinfusion enrichments were used to determine baseline isotope enrichments, which were subtracted from all subsequent measurements.

A rise-toward-plateau for plasma FC ^13^C enrichments was observed (Figure 2A). Although plateau was not achieved, plasma FC enrichments achieved on average 72% of the calculated plateau or asymptotic enrichment by the end of 32 hours. Infusions were performed in a subset of subjects (shown in Figure 2A). Plasma CE and RBC enrichments lagged behind the plasma FC enrichment curve. On the basis of the diminishing return obtained from the additional infusion time, the majority of subjects here (12 of the 17 total) were infused for 24 hours.

### Cholesterol Pool Sizes and Fluxes

Results from modeling cholesterol enrichments for 24 hours in all subjects are presented in Tables 1 and 2. TCE, calculated as the sum of [k(0,1)×xV_1_] and [k(0,3)×V_3_], was 3.79±0.88 mg/kg per hour (≍8 g/d). It should be noted that this flux parameter includes only nonfutile or net fluxes, which are defined by the model as irreversible loss of label from the rapidly exchanging FC pool. RBC–plasma exchange flux with plasma FC [k(1,2)×V2] was 3.38±1.10 mg/kg per hour. Because RBCs are not thought to actively efflux cholesterol to lipoproteins, this parameter could be taken as a metric representing the aqueous diffusion acceptor capacity of plasma.

**Table 2.** Cholesterol Flux and Rate Constants

		Flux, mg/kg per hour			Rate Constants, pools/hour			
Participants	TCE	Flux 1	Flux 3	Flux 2	k(0,1)	k(0,3)	k(1,2)	k(2,1)
435	3.73	3.07	0.67	1.14	0.047	0.016	0.080	0.015
442	4.68	3.86	0.82	4.08	0.064	0.022	0.163	0.056
454	3.72	2.89	0.83	3.15	0.047	0.017	0.126	0.040
458	3.46	2.75	0.71	3.86	0.054	0.019	0.155	0.061
459	3.30	2.69	0.60	3.47	0.050	0.019	0.139	0.052
58	5.29	4.15	1.14	5.19	0.083	0.022	0.226	0.082
63	4.21	3.24	0.97	1.51	0.068	0.020	0.069	0.024
64	2.40	1.34	1.06	3.12	0.016	0.019	0.127	0.021
61	3.80	2.73	1.07	2.82	0.054	0.019	0.077	0.040
65	5.31	3.95	1.36	2.62	0.058	0.019	0.076	0.029
66	4.35	2.30	2.05	3.13	0.047	0.032	0.108	0.034
69	3.92	2.98	0.94	2.30	0.069	0.018	0.077	0.041
70	2.79	1.68	1.11	4.35	0.045	0.023	0.077	0.071
71	3.26	2.29	0.97	4.43	0.077	0.015	0.078	0.105
72	2.80	1.01	1.79	4.33	0.040	0.024	0.081	0.063
73	2.70	1.33	1.42	4.67	0.042	0.018	0.087	0.075
75	4.69	3.53	1.16	3.22	0.047	0.019	0.102	0.032
Mean	3.79	2.69	1.10	3.38	0.053	0.0200	0.109	0.049
SD	0.88	0.94	0.38	1.10	0.016	0.0038	0.043	0.024

Individual rate constants for fluxes between pools presented in the model in Figure 1. Flux 1 indicates FC flux out of V1; Flux 3, CE flux (V3); Flux 2, flux from V2 (RBC FC) to V1; k(0,1), flux rate out of V1; k(0,3), flux rate out of V3; k(1,2), flux rate from V2 into V1; and k(2,1), flux rate from V1 to V2.

### Plasma FC Esterification Rate and CE Clearance Rate

Results are shown in Table 2. Mean CE formation and clearance rate was 1.10±0.38 mg/kg per hour. Esterification is therefore ≍1/8 of total flux into the plasma FC compartment, if RBC-derived FC flux is included, or ≍1/4 of irreversible flux into the plasma FC pool (ie, TCE)—that is, ≍1/4 of TCE goes to CE. Because the majority of LCAT activity is thought to occur on HDL, the plasma α-lipoprotein FC (instead of total plasma FC) enrichments were measured in 2 subjects, and the corresponding esterification rates were calculated by using SAAM II. For subject No. 69, the esterification rate constant was 1.7% per hour when fit to the plasma FC and 1.9% per hour when fit to the α-lipoprotein FC. For subject No. 70, the esterification rate was 2.4% per hour when fit to the plasma FC and 2.6% per hour when fit to the α-lipoprotein FC. Thus, values in these subjects were similar to but ≍10% higher than plasma FC when α-lipoprotein FC enrichments were used. Because LCAT esterifies cholesterol on both α- and β-lipoproteins,^39–40^ and because measuring α-lipoprotein FC enrichments involves more analytic steps and creates more possibility for error^30^ than measuring total plasma FC enrichments, the use of total plasma FC enrichments was the preferred approach.

### Effect of Intralipid Infusion on the Plasma Lipid Profile

There were no significant changes in lipoprotein distribution or total plasma concentrations of cholesterol over the course of the infusion of Intralipid/^13^C-cholesterol. Fast protein liquid chromatography analyses revealed no significant shift in the distribution of cholesterol among lipoprotein particles (not shown). Nuclear magnetic resonance analysis of particle size, size distribution, and number similarly was not changed in any systematic way (Table 3).

**Table 3.** Lipoprotein Particle Size During Infusion

	VLDL and Chylo Particles, nmol/L		LDL Particles, nmol/L		HDL Particles, μmol/L		VLDL Size, nm		LDL Size, nm		HDL Size, nm		TG (Total), mg/dL		VLDL and ChyloTG, mg/dL		HDL-C, mg/dL	
	Start	End	Start	End	Start	End	Start	End	Start	End	Start	End	Start	End	Start	End	Start	End
69	46	79	1141	680	25	20	68	40	21	22	8.9	8.8	125	90	91	68	35	27
70	17	42	759	925	18	18	56	50	21	21	9.5	9.5	38	59	17	37	31	31
72	83	50	1085	1756	21	26	45	64	21	20	8.4	8.8	107	132	78	99	26	35
73	45	39	1077	1134	26	24	44	50	22	21	9.4	9.4	77	89	41	57	42	40
77	22	22	756	876	38	44	86	55	21	22	9.1	9.0	100	64	72	31	60	69
78	32	46	986	1154	28	31	64	47	22	22	9.4	9.3	82	71	50	35	51	51
Mean	41	46	967	1088	26	27	61	51	21	21	9	9	88	84	58	55	41	42
SD	24	19	170	372	7	9	16	8	1	1	0	0	30	27	27	26	13	16

Lipoprotein particle distribution in subjects before the start (start) and during the 24th hour (end) of an intravenous infusion of Intralipid and ^13^C-cholesterol. Chylo indicates chylomicron; LDL, low-density lipoprotein; VLDL, very-low-density lipoprotein; and TG, triglyceride. There were no significant differences in any parameter by Wilcoxon matched-pairs test.

### Effect of Infusion Duration on the Modeling Parameters

We examined the effect of lengthening the modeled infusion time from ≍20 hours in 7 subjects who received a 32-hour infusion. Extending the data entered into the SAAM II model from 21 hours to 32 hours tended to reduce the calculated flux 1 by an average of 15% and flux 3 by an average of 3%.

### Equilibration Within Plasma α- and β-Lipoprotein Particles

We compared the enrichments in plasma β-lipoprotein particles precipitated by PEG6000 and the PEG6000 supernatant (α-lipoprotein particles) in plasma from several subjects. The β- and α-lipoprotein particle FC enrichment averaged 102±2% and 82±4%, respectively, of whole plasma FC at 20 hours. The modest gradient of dilution (α-lipoproteins slightly more dilute than β-lipoproteins) is consistent with the model^30^ that unlabeled FC from tissues enters the extracellular compartment first on α-lipoprotein particles and then mixes into the larger β-lipoprotein and hepatic FC pools. These results support the general finding of Schwartz et al^29^ that there is rapid equilibration of FC among lipoprotein particles in vivo in humans.

### Fecal Excretion Rate and Isotope Recovery

Neutral and acidic sterol excretion were measured in subjects after a 24-hour infusion of ^13^C_2_-cholesterol. Average neutral and acidic sterol excretion rates were 1430 and 430 mg/d, respectively (Table 4). Recovery of intravenously administered ^13^C_2_-cholesterol in fecal sterols was determined. Enrichment profiles of 2 representative subjects are shown (Figure 2B). The mass excretion and enrichment data were used to determine the fraction of isotope recovered over the 7-day period after intravenous [2,3-^13^C_2_]-cholesterol administration. This value represents the efficiency of plasma cholesterol excretion into fecal sterols over the course of 7 days. Recovery of neutral and acidic sterols averaged 12±4% and 7±3% of administered [2,3-^13^C_2_]-cholesterol, respectively (Table 4). Thus, ≍19% of the labeled FC infused into plasma was recoverable in fecal sterols over the course of 7 days.

**Table 4.** Neutral and Acidic Sterol Excretion and Recovery

	NS		BA	
Participants	mg/d	Percent ^13^C-Cholesterol Recovered	mg/d	Percent ^13^C-Cholesterol Recovered
435	831	19.7	320	5.0
442	1238	13.4	771	8.2
454	1471	12.1	771	4.9
458	992	16.6	441	6.0
459	761	9.4	417	3.5
58	1232	9.8	304	11.3
63	1449	13.2	343	11.4
64	737	6.8	236	10.7
61	1028	8.0	340	5.9
65	680	8.6	425	7.1
Mean	1042	12	437	7
SD	293	4	187	3

Individual rates of neutral and acidic sterol excretion in milligrams per day and percent ^13^C-cholesterol recovered over the 7 days after infusion.

## Discussion

The approach used here was designed on the basis of a large body of evidence, including previous models of the dynamics of whole-body cholesterol pools by pioneering investigators such as Schwartz, Goodman, Grundy, Dietschy, Ahrens, Zilversmidt, and others,^2,26,28,30,41–42^ as well as molecular characterization of the biology of the RCT pathway by many investigators.^6,12,42–43,45^

Several fundamental questions remain unanswered about the physiological functions of the various components of the systemic RCT pathway, as well as the relevance of systemic RCT fluxes to atherogenesis.^5–11,35^ In the present study, we set out to develop a clinical protocol that easily could be applied to larger studies and allowed dissection of the RCT pathway into fluxes through its modular components.^46^ We report that TCE was 3.79±0.88 mg/kg per hour, or ≍8 g/d, and that RBC FC–derived exchange flux into plasma FC was of a smaller though comparable magnitude (3.38±1.10 mg/kg per hour). Plasma FC esterification was 1.10±0.38 mg/kg per hour, or ≍28% of the net flux into the plasma FC pool. Recoveries were 7% and 12% of administered [2,3-^13^C_2_]-cholesterol in fecal BA and NS, respectively.

### Efflux and Pool Sizes

Of the 3 primary components of systemic RCT, the first step, efflux from cells into the extracellular compartment, is perhaps the most complex and least well understood. Previous investigators have identified whole-body cholesterol pools exhibiting a wide range of turnover rates.^25–27^ The majority of tissue FC exists in pools with half-lives of weeks or months, whereas other pools of FC have much more rapid turnover. Plasma FC is a pool that becomes isotopically equilibrated within hours, and plasma lipoprotein and hepatobiliary FC compartments are reported to be well mixed.^30^ The vast majority of cholesterol has been found to exit tissues into the plasma compartment in the form of FC, entering the rapidly equilibrated pool in blood, initially on HDL particles (see Schwartz et al^30^ and review^29^). We found that the non-RBC efflux rate, representing the movement of FC from extrahepatic tissues into and out of the rapid-turnover pool of FC that is sampled in the plasma compartment, was ≍8 g/d. In comparison, whole-body net cholesterol balances, which include dietary cholesterol absorption, de novo cholesterol synthesis, and fecal excretion pathways, are generally in the range of 1.0 to 1.5 g/d.^25,27,47^ Thus, the efflux rate of FC from tissues is 5 to 8 times the net outflow rate of sterols from the whole body.

It is of interest to compare previously published data to our calculated fluxes and pool sizes. Results from Schwartz et al are generally consistent^29–31^ with our findings but do show distinct quantitative differences. Their FC esterification rate of 114±28 mg/h was similar to our rate of 96±37 mg/h. Our estimate of FC flux from tissue to plasma, however, was 340±135 mg/h, which is higher than their value of 274±197 mg/h. The size of our total rapidly mixing pool (V1+V2) is ≍9.5±2 g, compared to average reported values of 25 g^26^ by radioactive decay. Given the difference in time scale (hours versus days) of the labeling methods, the rapidly mixed pool that we describe likely represents a portion of the previously described pool, after elimination of the contribution from “futile” (non-net) mixing.

Although the purpose of these studies was not to evaluate the relationship between plasma lipoprotein concentrations and cholesterol fluxes, we performed some exploratory Spearman nonparametric correlations between TCE and HDL-C (n=17). No significant trend was observed between HDL-C and TCE (r^2^=−0.03; *P*=0.52), which is consistent with the concept that HDL-C alone is an insufficient index of HDL function in vivo. Larger, prospectively designed studies need to be conducted to fully evaluate the relationship between HDL-C levels and cholesterol fluxes in vivo.

### LCAT Flux (Esterification)

Inclusion of LCAT flux in the model provides access to a unidirectional metabolic step and a potentially key therapeutic target. Esterification of FC in plasma is an energy-utilizing and irreversible step that can drive efflux from tissue cholesterol pools. Although LCAT activity was a central element in the original RCT concept,^14^ it still remains uncertain whether LCAT activation is antiatherogenic.^19^ The quantitative relationships that we observed between LCAT flux and the 2 other arms of systemic RCT proved to be of interest. Esterification rate was 1.10±0.38 mg/kg per hour, representing a quantitatively sizable disposal route for whole-body TCE. The effects of LCAT itself, as well as agents stimulating LCAT activity, are now accessible to experimental testing.

Compared to previous studies with radioactive tracers, our results for rate constants of FC to CE conversion, [k(0,3)], were 0.02±0.004 per hour, consistent with those from Nestel,^21,48^ Barter,^20,49^ and Schwartz,^28,30^ who reported values in the range of 0.01 to 0.04 per hour.

### Fecal Sterol Excretion

The canonical RCT pathway ends with excretion of fecal sterols via the hepatobiliary system, although recently a direct, plasma-to-stool transintestinal route has been proposed.^50^ Most drugs that increase HDL-C in humans have not been shown to increase net FSE, however, and the role of fecal excretion of sterols in the antiatherogenic actions of HDL remains uncertain. Moreover, flux from plasma cholesterol to fecal sterols must be differentiated from net FSE because hepatic de novo cholesterol synthesis and cholesterol absorption efficiency can be confounding factors. Previous measurements of the efficiency of excretion of plasma FC into fecal sterols over this period of time (7 days) have not, to our knowledge, been reported previously. It is useful to quantify NS and BA excretion separately, because the partitioning of hepatic secretion of cholesterol into biliary NS versus BA is influenced by the particle carrying the cholesterol (eg, α- versus β-lipoproteins) and the metabolic source (eg, FC versus CE).^51-53^

### Study Limitations

Some fundamental physiological and pathophysiological questions are not answered by this work. The first is whether energy-dependent FC efflux, facilitated efflux, and aqueous diffusion have similar functional consequences for cholesterol homeostasis. It is unlikely that, once mixed into the systemic FC pool, the fate of a cholesterol molecule depends on the route by which it got there. Because there is no such thing in membrane cholesterol biochemistry as true molecular “exchange” (eg, as with Na/K exchange transporters), FC molecules on HDL acceptor particles, regardless of their source, are equally capable of resulting in net (directional) outward flux or of returning to the tissue. The model presented here was designed to isotopically equilibrate and thereby remove from calculations the rapid mixing of FC in plasma lipoprotein pools that occurs early during labeled cholesterol infusions. Because of the long infusion times used here, it is reasonable to assume that the fluxes measured are not merely “futile” exchange but do indeed represent metabolically relevant events, but this assumption will require direct experimental validation.

A second question is whether systemic RCT is relevant to atherogenesis or antiatherogenic therapies. We measured systemic RCT fluxes on the basis of the assumption that the systemic capacity to mobilize, esterify, and excrete tissue cholesterol applies to atherogenic target tissues, including the vessel wall macrophage. Indeed, it might not be possible to measure the very small contribution from arterial wall macrophages to whole-body cholesterol dynamics.

It is worth emphasizing that experimental resolution of these basic questions about the relationship between atherogenesis and arms of the RCT pathway requires the capacity to measure RCT fluxes in vivo.

In summary, we describe a technique for quantifying in vivo the flux rates through 3 key components of the systemic RCT pathway. Several conclusions can be drawn. Whole-body efflux rates can be measured in humans by a constant [2,3-^13^C_2_]-cholesterol infusion approach that includes measurements of FC exchange with RBC, esterification of plasma FC, and clearance of CE. Flux from plasma cholesterol into fecal sterols can be determined by collection of stool samples and use of an oral sitostanol administration protocol. Many unanswered questions about RCT and the role of HDL in this pathway now can be addressed by use of this approach.
